# Identification and characterization of QTL for grain protein content derived from the D genome of allohexaploid wheat

**DOI:** 10.3389/fpls.2025.1711891

**Published:** 2025-12-01

**Authors:** Xinghua Luo, Tianjiao Shao, Shanshan Zhai, Xinhao Meng, Shidian Wen, Chaojie Xie, Mingshan You, Rongqi Liang, Zhongfu Ni, Qixin Sun, Runqi Zhang, Baoyun Li

**Affiliations:** 1 State Key Laboratory of High-Efficiency Production of Wheat-Maize Double Cropping, China Agricultural University, Beijing, China; 2 Beijing Academy of Agriculture and Forestry Sciences, Beijing, China; 3 Chinese Academy of Agricultural Sciences, Beijing, China

**Keywords:** wheat, grain protein content (GPC), QTL, fine mapping, wheat quality

## Abstract

**Introduction:**

Identifying and utilizing major quantitative trait loci (QTLs) related to wheat grain protein content (GPC) is critical for the wheat quality improvement. However, the identification of genes regulating GPC remains relatively limited.

**Methods:**

In this study, a genetic population containing 198 recombinant inbred lines (RILs), derived from a cross between common allohexaploid wheat cultivar TAA10 and synthetic allohexaploid wheat cultivar XX329 was used to identify QTLs associated with GPC.

**Results:**

Three major QTLs for GPC were detected on chromosomes 2D, 4D, and 7D (*Qgpc.cau-2D*, *Qgpc.cau-4D*, and *Qgpc.cau-7D*) respectively. Among them, *QGpc.cau-2D* located between markers *Xbarc124* and *2S178*, exhibited the largest additive effect and explained 4.70-17.97% of the phenotypic variation. Using progenies from the key residual heterozygous line124, *QGpc.cau-2D* was fine mapped to an approximately 4.2 Mb physical interval between the markers *Xcau-2D541* and *Xcau-2D781*. According to the analysis of genes within the interval, *TraesCS2D03G0079200*, *TraesCS2D03G0080700*, *TraesCS2D03G0081400*, and *TraesCS2D03G0088900* were predicted as putative candidate genes.

**Discussion:**

These results provide a foundation for the cloning of candidate genes related to GPC and the genetic improvement of wheat quality.

## 1 Introduction

Bread wheat (*Triticum aestivum* L.) is a major global grain crop, supplying over 20% of human dietary calories and protein (Shiferaw et al., 2013). Wheat grain proteins are broadly categorized into non-gluten and gluten protein. Non gluten protein includes water-soluble albumins and salt-soluble globulins, while the gluten protein is primarily composed of gliadin and glutenin. Gliadin and glutenin constitute up to 85% of the total grain protein content (GPC) in wheat grain. Unlike other Gramineae crops, wheat flour contains gluten proteins that enable the production of diverse food products such as bread, noodles, and pastries (Rustgi et al., 2019). The gluten content is closely related to GPC and plays a crucial role in determining wheat processing quality (Barak et al., 2015; Ma et al., 2019).

Several methods are commonly used to evaluate the processing quality of wheat flour, including GPC, gluten index, SDS-sedimentation value (SSV), wet gluten content (WGC), and dough rheology. GPC is a primary determinant of end-product quality and is widely adopted as a key criterion for wheat quality assessment (Blanco et al., 2006b). Meanwhile, GPC is strongly correlated with various wheat quality and yield traits, Oelofse et al (Oelofse et al., 2010). reported that SSV was significantly positively correlated with GPC and mixograph development time. Furthermore, substantial evidence indicates negative correlations between GPC and both grain starch content (GSC) and thousand grain weight (TGW) (Blanco et al., 2012; Guo et al., 2023).

The GPC of wheat is controlled by multiple genes, known as quantitative trait loci (QTLs). Previous studies have detected GPC-associated QTLs on nearly all chromosomes of bread wheat (Dholakia et al., 2001; Blanco et al., 2006a). For instance, QTLs for GPC were identified on chromosomes 1A, 1B, 2A, 2B, 5B, 6B and 7A using an doubled haploid (DH) population derived from the cross DT695×Strongfield (Suprayogi et al., 2009). Two major GPC QTLs were mapped on chromosomes 1B and 6A using 187 DH lines derived from the cross between cvs. Courtot and Chinese Spring (Perretant et al., 2000). Genes associated with GPC, such as *Gpc-B1*(*NAM-B1*), have been cloned (Uauy et al., 2006). The *Gpc-B1* mutant resulted in a reduction of GPC, while its effect on carbohydrate accumulation in wheat grains was not significant (Avni et al., 2014). Moreover, *TaAAP6-3B*, a regulatory factor for GPC has also been identified (Jin et al., 2018). *TaGW2* mutants demonstrated significantly higher TGW and GPC compared to the wild type, broking the negative correlation between grain yield (GY) and quality traits in wheat (Zhang et al., 2018). Expression of the 1Ay21* gene has the potential of simultaneously increasing GPC and GY under certain environment (Roy et al., 2020). Collectively, while numerous QTLs for GPC have been preliminarily mapped, with few of them focusing on the fine mapping. Meanwhile, very few GPC genes have been cloned due to the complexity of the wheat genome (Zhao et al., 2021). It is necessary to fine mapping the GPC candidate genes that provide gene resources for wheat quality improvement.

It is well konwn that allohexaploid wheat (*Triticum aestivum*) (genomes AABBDD) originated via hybridization of tetraploid wheat (*Triticum turgidum*) (genomes AABB) and diploid goat grass (*Aegilops tauschii*) (genomes DD) (Dvorak et al., 1998, 2012). It has been demonstrated that *Aegilops tauschii* offers a rich source of allelic diversity on wheat quality and yield to D-genome of common wheat (Yu et al., 2014; Kou et al., 2023). Notably, a unique set of *Glu-D1* allelic variations in *Aegilops tauschii*, such as *1Dx1.1^t^* (Fang et al., 2009), distinction from those present in current wheat germplasm has been identified (Delorean et al., 2021). This suggests that the D subgenome of *Aegilops tauschii* may harbor numerous genes associated with wheat quality traits.

In this study, a recombinant inbred line (RIL) population comprising 198 lines derived from a cross between TAA10 and XX329 was used to identify QTL for GPC. TAA10 is a Canadian high-gluten wheat cultivar, whereas XX329 is a synthetic allohexaploid wheat obtained by crossing an allotetraploid wheat derived from TAA10 with *Aegilops tauschii* subsp. *strangulata* (line TQ18, DD). TAA10 and XX329 exhibited 96.55%, 98.10%, and 66.26% genetic similarity in the A, B, and D genomes, respectively (Zhang et al., 2014; Yan et al., 2017). Three QTLs associated with GPC were mapped on chromosomes 2D, 4D, and 7D (*Qgpc.cau-2D*, *Qgpc.cau-4D*, and *Qgpc.cau-7D*). Based on the results of the primary mapping, the residual heterozygous lines of *Qgpc.cau-2D* from the RIL population were selected for self-pollination, and a secondary segregating population was constructed to fine map *Qgpc.cau-2D*. The aim of the study was to explore and utilize the genes or beneficial allelic variants controlling GPC, providing a foundation for the subsequent cloning of GPC candidate genes and genetic improvement of wheat quality.

## 2 Materials and methods

### 2.1 Plant materials and field trials

The RIL population was developed from a cross between the Canadian common wheat cultivar TAA10 and the resynthesized allohexaploid wheat line XX329. The RIL population and its parental lines were planted in Beijing (40°N, 116°E), Handan (Hebei Province, 36°N, 114°E), and Shijiazhuang (Hebei Province, 38°N, 115°E) from 2017 to 2020 (**Supplementary Table S1**). The residual heterozygous lines RIL-124 and RIL-144 from the RIL population, along with their derived progeny, were used for *Qgpc.cau-2D* fine-mapping. The progeny of RHL-124 and RHL-144 were planted in Beijing in the fall of 2021, and individual plants with recombination (124-8, 124-11, 124-35, 124-61, and 124-164) were planted in Hebei in the fall of 2022. New recombinant lines (124-61-97, 124-164-161, 124-164-9, and 124-164-181) were cultivated in Hebei during the fall of 2023. A randomized block design was adopted in six environments, with three replications. There were two rows for each replicate of RILs. The rows were 1.5-m long and 30-cm apart, with 20 seeds sown in each row. Materials planted in individual were sown 15 seeds in each row. All management of field trials was consistent with local standard practice.

### 2.2 Phenotypic identification and statistical analysis

Grain protein content (GPC), wet gluten content (WGC), test weight (TW), grain hardness (GH), and grain starch content (GSC) were determined using a near-infrared grain analyzer (Perten DA 7200). The SDS-sedimentation value (SSV) was measured according to a previously published method (Axford et al., 1979).

Basic statistical analysis was conducted using SPSS version 20.0. R software v3.6.2 was used to calculate Best Linear Unbiased Prediction values (BLUP), conduct Shapiro-Wilk tests, and generate frequency density histograms for wheat quality traits. The significance of differences was determined by Student’s *t* test. The broad-sense heritability ( $H_{B}^{2}$) was calculated by the following formula:


HB2=σg2(σg2+σe2n)

where $\sigma_{g}^{2}$ was estimated genetic variance, $\sigma_{e}^{2}$ was the residual error variance, *n* was the number of environments (Xu et al., 2017).

### 2.3 QTL analysis

The genetic linkage map was generated using JoinMap 4.0, RECORD 2.0, and Windows QTL Cartographer Version 2.5 (Van Os et al., 2005; Ooijen et al., 2006) as described by Xu et al. (2022). QTL analysis was performed using composite interval mapping in Windows QTL Cartographer Version 2.5, with GPC values from individual environments and their corresponding BLUPs serving as phenotypic inputs (Wang et al., 2005) (**Supplementary Tables S2**, **S3**). A threshold LOD value was determined for each of the trials applying the permutation program that was run repeatedly for 1000 times at P≤ 0.05. The threshold value varied with different trials, ranging from2.3 to 2.5. To be more precise, 2.5 was used as the threshold for all trials. The confidence interval of each QTL was defined as the peak location ± 2 LOD. Overlapping confidence intervals among several QTLs were initially regarded as a single QTL. A QTL was considered environmentally stable if detected in more than two environments.

### 2.4 Marker development and genotyping

The genomic DNA was extracted from fresh leaf tissue using the cetyltrimethyl ammonium bromide (CTAB) method (Allen et al., 2006). Publicly available simple sequence repeat (SSR) and insertion/deletion (InDel) markers were employed to genotype variant loci associated with GPC for QTL analysis. The primer sequences for most of the public SSR markers were sourced from https://wheat.pw.usda.gov/GG3/. InDel fragments longer than 6 bp within the candidate region for the GPC QTL were identified by comparing resequencing data of TAA10 and XX329. The 200-bp flanking sequences of each InDel were retrieved from the Wheat Multi-Omics Database (wheatomics.sdau.edu.cn). Primers were designed with Primer3.0 (https://bioinfo.ut.ee/primer3-0.4.0/). The primer sequences of InDel markers used in this study are listed in the **Supplementary Table S6**. The PCR mixture (10 µL total volume) contained 1 μl DNA template (concentration of 50–100 ng), 2 μl primer (mixture of left and right primer, 2 μM), 5 μl 2 × Taq PCR StarMix and 2 μl of ddH_2_O. The PCR program was performed as follows: 94°C 5 min; 35 cycles of 94°C30 s, 57°C 30 s, 72°C 30 s; 72°C 10 min. An 8% non-denatured polyacrylamide gel electrophoresis (PAGE) was used for fragment length analysis (Marklund et al., 1995).

### 2.5 Candidate genes analysis

Using Triticeae Gene Tribe platform (http://wheat.cau.edu.cn/TGT/index.html) (Chen et al., 2020) and Wheat Multi-Omics Database, genes within the target interval for potential effects on GPC were screened, based on gene function annotations and expression profile in Chinese Spring. Resequencing data facilitated sequence analysis of the TAA10 and XX329. Additionally, amino acid sequences were compared to identify missense mutations.

The grains at 15, 20, 25, and 30 days post-anthesis (DPA) of TAA10 and XX329 were collected for RNA extraction. Total RNA was extracted from grains using TRIzol (Invitrogen) method. cDNA was synthesized from RNA using a reverse transcription kit (TaKaRa, code RR047A) for quantitative real-time PCR. TaActin was used as an internal control. The PCR protocol comprised an initial denaturation step at 95°C for 5 min, followed by 40 cycles of 95°C for 15 s (denaturation), 60°C for 20 s (annealing), and 72°C for 20 s (extension), with a subsequent melting curve analysis. All qRT-PCR experiments were performed with three independent biological replicates. The relative fold changes were calculated using the comparative CT method (2 ^−ΔΔCT^).

## 3 Result

### 3.1 Phenotypic evaluation

**Figure 1:**
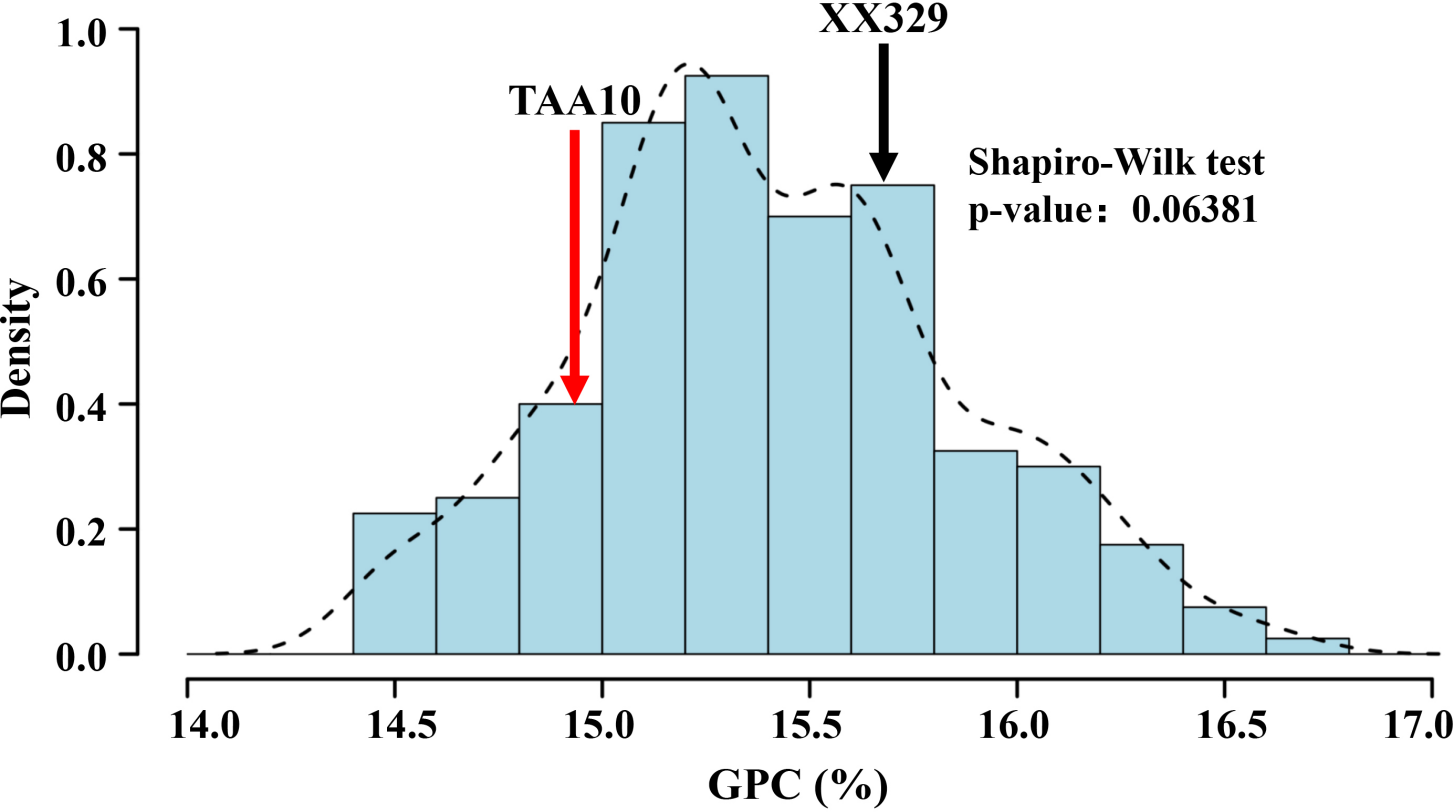
The frequency distribution histograms for grain protein content (GPC) of the RIL population from TAA10/XX329. The *Y-axis* represents the density (the ratio of frequency to group distance) of GPC. *P* < 0.05 indicates a significant departure from the normal distribution (Shapiro-Wilk test).

GPC was determined for the parental lines (XX329 and TAA10) and all RILs population across multiple environments. The GPC of XX329 ranged from 13.68% to 17.40%, whereas TAA10 exhibited a range of 13.72% to 15.87% (**Table 1**). XX329 showed higher GPC than TAA10 in five environments. Frequency distribution analysis revealed significant bidirectional transgressive segregation for GPC in the RIL population across all environments. The Shapiro-Wilk normality test demonstrated that the BLUPs for GPC were normally distributed (*P* > 0.05), which indicated that GPC is a quantitative trait controlled by multiple genes (**Table 1**, **Figure 1**). Analysis of variance indicated that environmental effects accounted for the largest proportion of GPC variation (54.5%), followed by genetic effects (25.7%) and genotype × environment interaction (19.8%). Despite this, the broad-sense heritability estimate was 0.82, demonstrating that GPC remains predominantly under genetic control after accounting for environmental variance (**Table 2**).

**Table 1:** Statistical analysis of GPC in RIL population, TAA10 and XX329 under different environments.

Trait	Environment	TAA10	XX329	RIL			BLUP
		Mean	Mean	Mean	Minimum	Maximum	
GPC (%)	E1	14.68	15.03	15.05	13.04	17.17	15.4
	E2	15.03	17.40	16.33	11.51	18.91
	E3	15.87	17.27	16.59	14.96	18.25
	E4	14.83	15.40	15.47	13.89	17.73
	E5	13.72	13.68	14.01	12.05	16.25
	E6	14.38	15.64	14.96	13.64	16.89

GPC presented grain protein content.

**Table 2:** Analysis of variance of GPC and broad-sense heritability in RIL population.

Trait	Source of variation	DF	Sum of Squares	%TSS
GPC	Block/environment	6	–	–
	Genotype (G)	197	1291.61	25.7
	Environment (E)	5	2734.85	54.5
	G × E	985	992.16	19.8
	GVC (%)	16.5	–	–
	*H^2^_B_*	0.82	–	–

GVC presented genetic coefficient of variation; DF presented degree of freedom; %TSS presented percentage relative to total sum of squares.

### 3.2 The impact of grain protein content on quality-related traits

To investigate the effects of GPC on other quality traits, we performed Pearson correlation analysis on BLUP of quality parameters in RIL population using IBM SPSS software. Correlation analysis revealed a highly significant positive relationship between GPC and WGC. Conversely, GPC and WGC showed significantly negative correlations with GSC and TW. No significant correlations were detected between GPC and GH or SSV. Significant interrelationships among other traits were also observed (**Table 3**).

**Table 3:** The correlation between GPC and other quality‐related traits under different environments.

BLUP	GPC	GSC	WGC	GH	TW
GSC	-0.92**				
WGC	0.97**	-0.94**			
GH	0.11	-0.33**	0.28**		
TW	-0.53**	0.75**	-0.63**	-0.55**	
SSV	-0.08	-0.05	-0.04	0.30**	-0.17*

GPC presented grain protein content; GSC presented grain starch content; WGC presented wet gluten content; TW presented test weight; GH presented grain hardness. The values represent Pearson’s correlation coefficients. Asterisks indicate significant differences (*P < 0.05, **P < 0.01).

### 3.3 QTL analysis of wheat grain protein content

**Figure 2:**
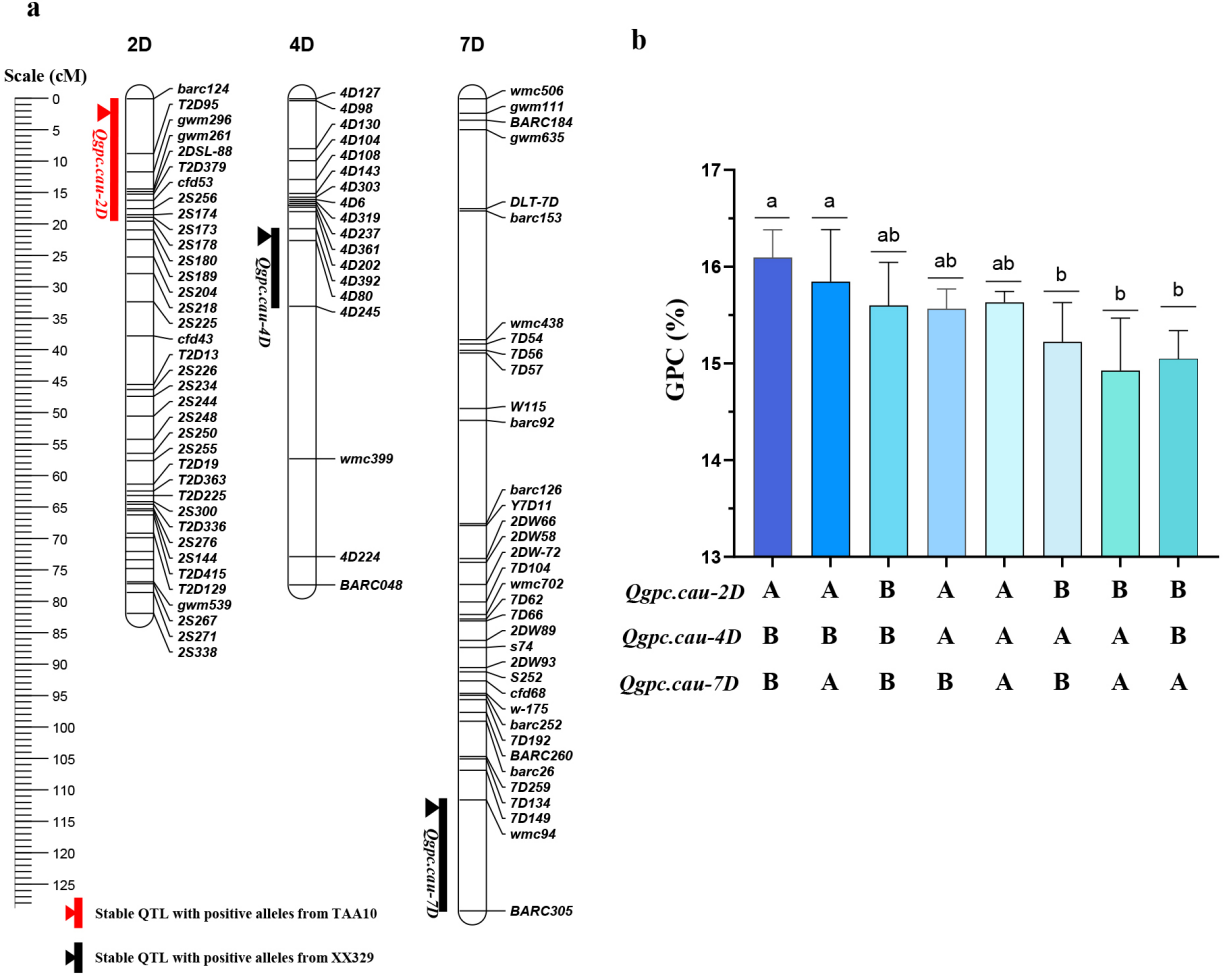
Primary QTL mapping for GPC by genetic map. **(a)** Genetic locations of GPC related QTL regions associated with GPC. Environmentally stable QTL with positive alleles from TAA10 and XX329 were indicated with red and black bars and triangles, respectively. **(b)** The polymerization effect of *Qgpc.cau-2D*, *Qgpc.cau-4D*, and *Qgpc.cau-7D* on GPC in the RIL population. A and B represent the genotypes of TAA10 and XX329, respectively. Different letters indicate significant differences at the *P* < 0.05 level.

Based on the D genome genetic linkage map constructed by Xu et al (Xu et al., 2022). (**Supplementary Table S3**), together with genotypic and phenotypic data from RIL populations across six environments and BLUP values, we identified three QTLs associated with GPC on chromosomes 2D, 4D, and 7D (*Qgpc.cau-2D, Qgpc.cau-4D*, and *Qgpc.cau-7D*). *Qgpc.cau-2D* could be detected in four environments and BLUP values, with LOD score ranging 2.61-8.41. The positive allele of *Qgpc.cau-2D* was derived from TAA10, which explained 4.70%-17.97% of the GPC variation. This locus was located between the makers *barc124* and *2S178. Qgpc.cau-4D* explained 7.22%-11.59% of GPC variation and the positive alleles of this QTL was provided by XX329. This locus was detected across two environments and BLUP values, exhibiting LOD score ranging 3.15-4.56, with its physical position flanked by markers *4D130* and *4D245*. The positive allele of *Qgpc.cau-7D* was derived from XX329, which explained 6.83%-10.84% of the GPC variation. This QTL was detected across three environments and BLUP values, exhibiting LOD scores of 2.67-3.21, and was mapped to the *wmc94* and *BARC305* interval (**Table 4**, **Figure 2a**).

**Table 4:** QTLs for the GPC detected in all environments in the RIL population.

QTL	Maker interval	LOD	Add	PVE (%)	Environments
*QGpc.cau-2D*	*barc124-2S178*	2.61-8.41	-0.17~-0.29	4.70-17.97	E3, E4, E5, E6, BLUP
*QGpc.cau-4D*	*4D130-4D245*	3.15-4.56	0.16~0.25	8.32-12.65	E3, E4, BLUP
*Qgpc.cau-7D*	*wmc94-BARC305*	2.67-3.21	0.14 ~ 0.21	6.83-10.84	E3, E4, E6, BLUP

Add, additive effect of QTL; PVE (%), the phenotypic variation explained by QTL; “-” represents positive alleles from TAA10; “+” represents positive alleles from XX329.

In addition, some of the lines were homozygous at the loci of *Qgpc.cau-2D*, *Qgpc.cau-4D*, and *Qgpc.cau-7D* in the RIL population, which formed differentially combined genotypes. The result revealed that genotypic combinations harboring positive alleles derived from TAA10 and XX329 exhibited the highest GPC (**Figure 2b**), suggesting the additive effects among *Qgpc.cau-2D*, *Qgpc.cau-4D*, and *Qgpc.cau-7D*, which could be used for gene pyramiding breeding.

### 3.4 Fine mapping and verification of the *Qgpc.cau-2D*

**Figure 3:**
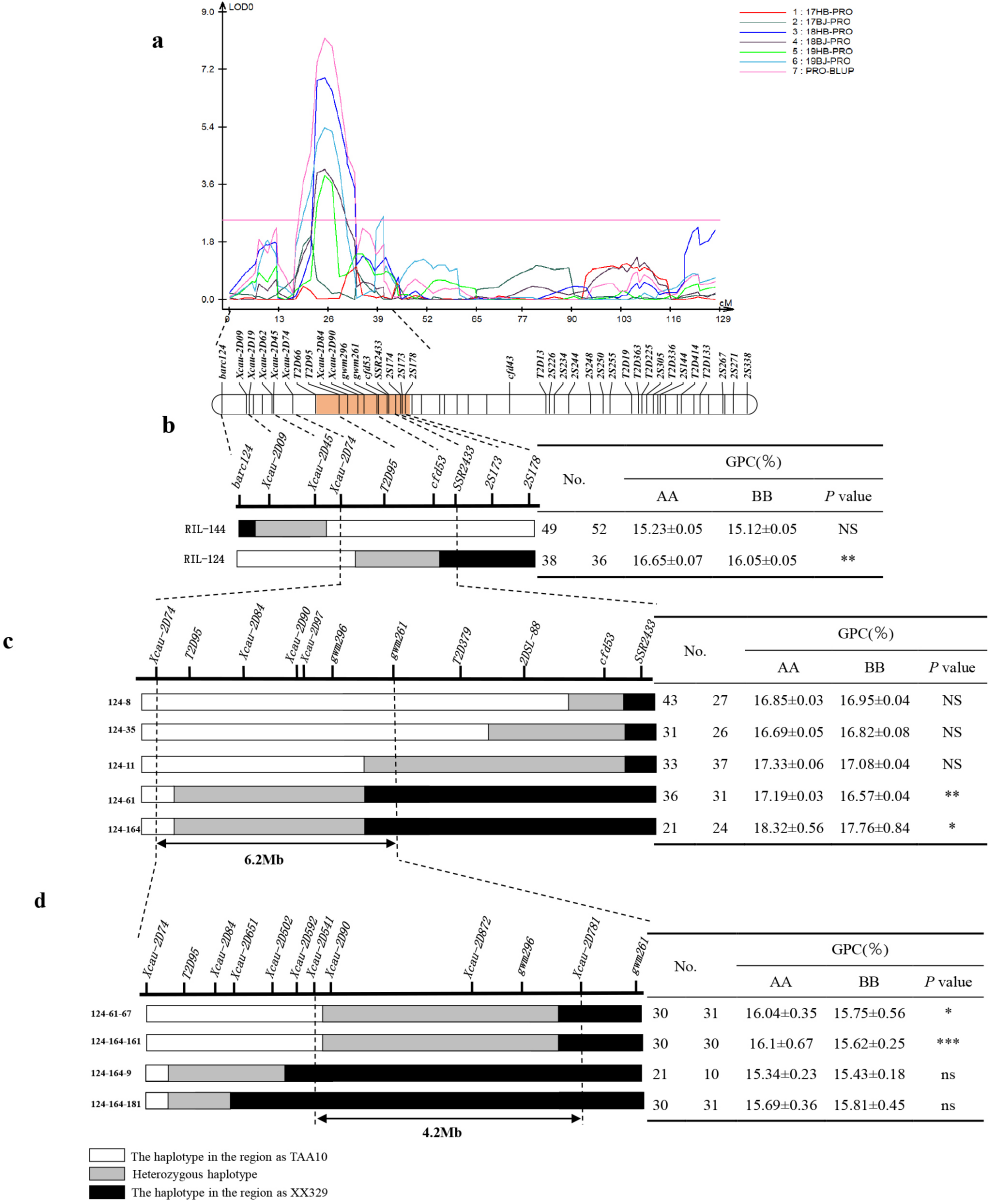
Fine mapping of the *Qgpc.cau-2D*. **(a)** QTL mapping for GPC in six environment and BLUP values. **(b)** Genotypic and phenotypic analysis of residual heterozygous lines RIL124 and RIL144, we narrowed the candidate interval to the genomic region between markers *Xcau-2D74* and *SSR2433*. **(c)** Genotypic and phenotypic analysis of five recombinant individuals (124-8, 124-11, 124-35, 124-61, and 124-164) enabled the refinement of the candidate region to the interval between markers *Xcau-2D74* and *gwm261*. **(d)** Genotypic and phenotypic analysis of four recombinant lines (124-61-67, 124-164-161, 124-164-9, and 124-164-184) delineated the candidate region to the interval between markers *Xcau-2D541* and *Xcau-2D781*. Left side is markers used to screen recombinants and the graphical genotypes of recombinants. Right side is the comparisons of GPC between TAA10 (AA) and XX329 (BB) within each family. No.: indicates the number of single plants with homozygous (TAA10/XX329) genotypes in the segregating population of progeny; data were analyzed using *Student's* t-test, asterisks indicate significant differences (*P < 0.05, **P < 0.01).

Since *QGpc.cau-2D* exhibited the highest additive effect and the largest proportion of phenotypic variation for GPC, it was prioritized for fine mapping. To fine map *QGpc.cau-2D*, we developed 13 InDel markers, increasing the total marker count on chromosome 2D from 43 to 56, thereby expanding the genetic map length of 2D from 81.59 cM to 129.13 cM, and improving the average marker density of 2.30 cM (**Supplementary Tables S4**, **S5**). The candidate region of *QGpc.cau-2D* was mapped to an interval of approximately 24 Mb, defined by markers *barc124* and *2S178* (**Figure 3a**).

To narrow the genetic interval of *QGpc.cau-2D*, we selected residual heterozygous lines RIL144 and RIL-124 from the RIL population. The GPC of segregating populations derived from the line RIL-124 and RIL144 were analyzed. The results showed that the lines with TAA10 genotype derived from RIL-124 exhibited significantly higher GPC values than the lines with XX329 genotype. Collectively, we delimited *QGpc.cau-2D* to the interval flanked by markers *Xcau-2D74* and *SSR2433* (**Figure 3b**). Five different types of heterozygous recombinant plants (124-8, 124-11, 124-35, 124-61, and 124-164) were selected. Student’s *t-*test analysis indicated no significant difference in GPC between TAA10 and XX329 genotypes in the segregating populations of lines 124-8, 124-11, and 124-35. In contrast, TAA10 and XX329 genotypes derived from 124–61 and 124–164 lines exhibited significant difference in GPC. Combining the genotype and phenotype data, the candidate region of *QGpc.cau-2D* was narrowed into a physical interval of 6.2Mb flanked by the markers *Xcau-2D74* and *gwm261* (**Figure 3c**). Subsequently, key recombinant individuals 124-61–67 and 124-164–161 were selected. The GPC of TAA10 genotypes was significantly higher than that of XX329 genotypes in two segregating populations (124-61–67 and 124-164-161). Finally, *QGpc.cau-2D* was delimited to the interval between molecular markers *Xcau-2D541* and *Xcau-2D781*, corresponding to the physical interval of 4.2 Mb according to the IWGSC RefSeq v2.1(**Figure 3d**).

To validate genetic effects of *QGpc.cau-2D* in wheat breeding, one specific STARP marker (*Xcau-2D612*) was developed based on the SNP in the candidate region and used to genotype the 198RILs plants with known GPC phenotypes. The result showed 74RILs possessing TAA10 alleles(C) had an average GPC value of 15.55%, and 108RILs possessing XX329 alleles(A) had an average GPC value of 15.31%, representing a statistically significant difference (**Supplementary Figure S1**). The marker *Xcau-2D612* showed co-segregation with the quantitative trait locus *QGpc.cau-2D*. This finding holds promise for future marker-assisted breeding to develop wheat varieties with elevated GPC.

### 3.5 Candidate genes analysis in the *Qgpc.cau-2D* region

Based on the positions of molecular markers *Xcau-2D541* and *Xcau-2D781*, the chromosome interval of locus *QGpc.cau-2D* was between 16002898 and 20194247 bp on the Chinese Spring reference genome sequences v2.1 (IWGSC, http://www.wheatgenome.org/). The results showed that there were 178 genes in the physical interval, including 78 high-confidence genes (*TraesCS2D03G0079100* to *TraesCS2D03G0096600*) and 100 low-confidence genes (*TraesCS2D03G0079300LC* to *TraesCS2D03G0096800LC*) (**Supplementary Table S7**).

**Figure 4:**
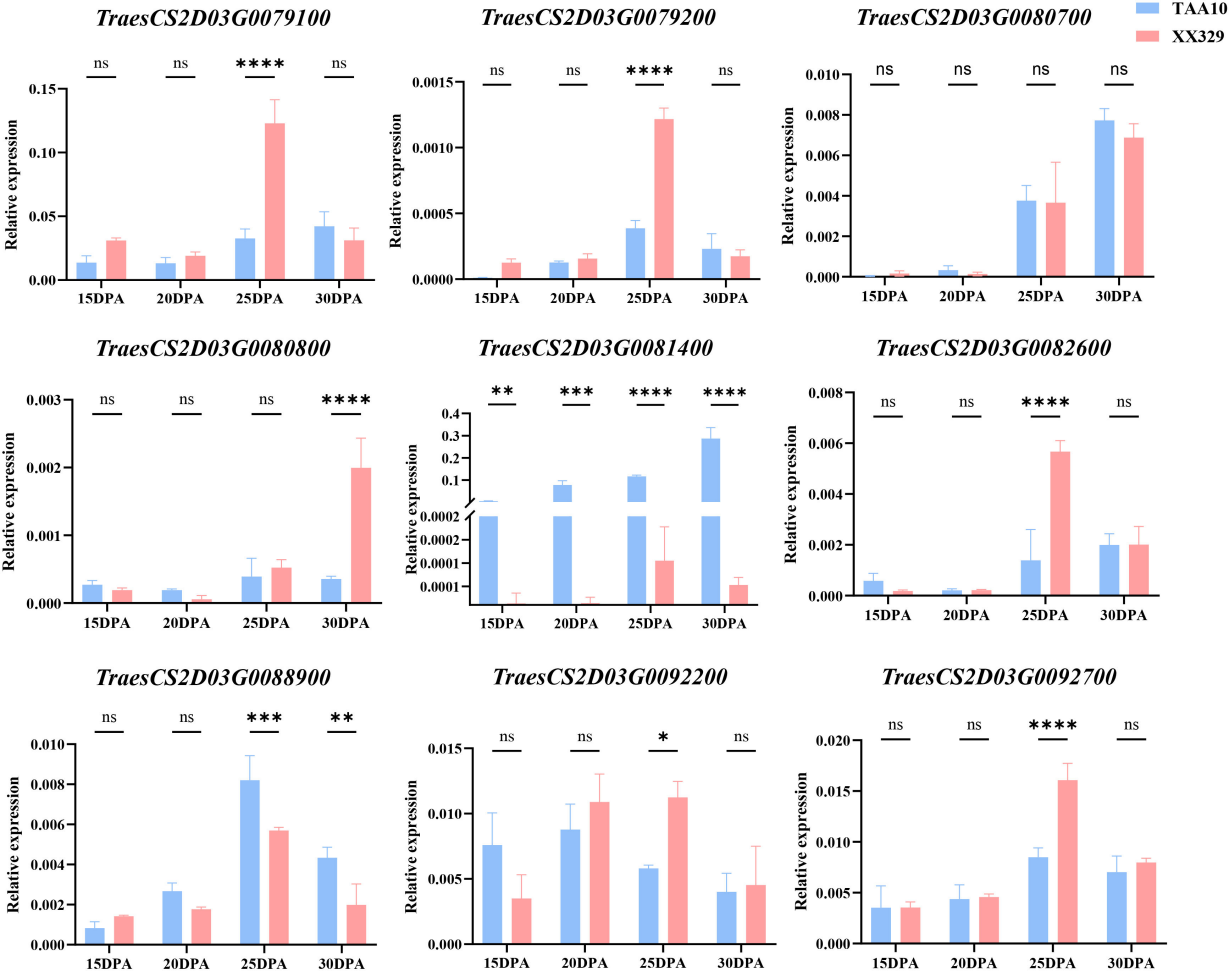
Expression validation of high-confidence genes located in the *Qgpc.cau-2D* -containing interval by qRT–PCR. Values are means ± SE from three independent biological replicates, and were analyzed using *Student's* t-test. *TaActin* was used as an internal control. Asterisks indicate significant differences (*P < 0.05, **P < 0.01).

Moreover, the expression profile of those high-confidence genes were investigated on the Wheat eFP Browser (https://bar.utoronto.ca/efp_wheat/cgi-bin/efpWeb.cgi) (Ramírez-González et al., 2018). Among 78 high-confidence genes, 20 were expressed in developing wheat grains. Comparative sequence analysis of the parental lines identified 12 genes harboring SNP or InDel polymorphisms, which were selected for further investigation. *TraesCS2D03G0080700* and *TraesCS2D03G0081400* encode indole-2-one monooxygenases. *TraesCS2D03G0080700* harbors three SNPs between TAA10 and XX329. Only the SNP located in exon 1 induces an amino acid substitution. *TraesCS2D03G0081400* harbors three SNPs that result in amino acid change. Although nucleotide variations exist in ten genes (*TraesCS2D03G0079100*, *TraesCS2D03G0079200*, *TraesCS2D03G0080800*, *TraesCS2D03G0082600*, *TraesCS2D03G0084300*, *TraesCS2D03G0084900*, *TraesCS2D03G0088900*, *TraesCS2D03G0089000*, *TraesCS2D03G0092200*, and *TraesCS2D03G0092700*) between TAA10 and XX329, all mutations are synonymous (**Supplementary Table S8**). We selected genes harboring variations in the promoter regions and missense mutations in the coding sequences of both parental lines to assess their relative expression in grains of TAA10 and XX329 at 15–30 DPA. The relative expression level of *TraesCS2D03G0081400* was continuously higher during the period of 15–30 DPA in TAA10 than that of XX329. *TraesCS2D03G0088900* showed significantly higher expression levels in TAA10 grains during the 25-30DPA compared to XX329. An additional six genes (*TraesCS2D03G0079100*, *TraesCS2D03G0079200*, *TraesCS2D03G0080800*, *TraesCS2D03G0082600*, *TraesCS2D03G0092200*, and *TraesCS2D03G0092700*) exhibited significantly higher expression levels in XX329 grains during the late grain-filling stage compared to TAA10. We hypothesize that these genes may function in the negative regulation of GPC. The relative expression levels of *TraesCS2D03G0080700* in developing grains showed no significant differences between TAA10 and XX329 at 15–30 DPA (**Figure 4**). These putative candidate genes will require further fine-mapping and functional validation via transgenic approaches.

## 4 Discussion

GPC plays a key role in determining both the nutritional and processing qualities of wheat flour, highlighting its importance in the wheat genetic improvement. Previous studies have identified numerous GPC related QTLs on the A and B sub-genomes (Suprayogi et al., 2009; Saini et al., 2022; Li et al., 2024). The RIL population derived from TAA10/XX329 in this work exhibits predominant variation in the D sub-genome, providing more opportunities for the exploration and utilization of genetic resources within D sub-genome.

Varieties with low GPC may contained some positive-effect allele of GPC. The lines with expected high GPC can be developed by pyramiding these positive-effect alleles (Jiang et al., 2021). In this study, XX329 showed higher GPC than TAA10. However, the positive-effect allele of *QGpc.cau-2D*, *Qgpc.cau-4D*, and *Qgpc.cau-7D* were derived from different parental lines. Furthermore, RILs pyramiding positive-effect alleles of GPC demonstrated high GPC (**Figure 2b**), consistent with previous studies (Li et al., 2009; Fatiukha et al., 2020). Therefore, it is more feasible to improve GPC for exploring and polymerizing positive-effect alleles in local varieties (Jiang et al., 2021).

Protein and starch constitute the primary storage reserves in wheat grains, collectively serving as the primary determinants of end-use quality for wheat food products. In this study, GPC and WGC exhibited highly significant negative correlations with GSC (**Table 3**). Increased starch accumulation in wheat grains tends to reduce GPC and WGC, suggesting energy competition between protein and starch biosynthesis during grain filling (Guo et al., 2023). Conversely, GPC exhibited a strong positive correlation with WGC, consistent with prior research (Ma et al., 2014, 2019). Additionally, SSV showed no significant correlations with GPC or WGC. Whereas Oelofse et al. (2010). reported a significant positive correlation between SSV and GPC, Zhai et al. (2024). found no such association. Consequently, the relationship between these traits warrants further investigation.

According to this study, the corresponding physical region of *Qgpc.cau-7D* in the Chinese Spring reference genome v2.1 spanned from 531to 569 Mb. *Qgpc.cau-4D* in this study corresponded to an approximate 419 Mb physical interval on the Chinese Spring reference genome v2.1. Previous research has also identified QTLs related to GPC on chromosome 4D (Groos et al., 2003; Guo et al., 2020), including the co-localization of *QGpc.yaas-4D* with *Rht-D1* (Hu et al., 2024). Additionally, another GPC related QTL have been co-localized with multiple quality-related traits on chromosome 4D (Mann et al., 2009). Due to the large size of the two candidate intervals, it remains unclear whether they constitute novel, previously unreported loci or represent allelic variants of known genes. Fine-mapping of these regions is necessary to resolve this issue. The *QGpc.cau-2D* in this study exhibited the highest additive effect and phenotypic contribution. We identified key residual heterozygous lines RIL124 and RIL144, and subsequent phenotypic analysis revealed significant differences in GPC among the segregating progeny of RIL124. This enabled us to initially delineate the candidate region between markers *Xcau-2D74* and *SSR2433*. The candidate region was ultimately narrowed to the interval flanked by markers *Xcau-2D541* and *Xcau-2D781* through fine-mapping. This locus corresponded to an approximate 4.2 Mb physical interval on the Chinese Spring reference genome v2.1. Laperche et al (Laperche et al., 2007). identified seventeen QTLs on genome 2D1-2 (QTL located near marker gwm484) region through their investigation of the genetic basis of nitrogen use-efficiency and genotype × nitrogen interactions. These QTLs were associated with nitrogen harvest index, straw nitrogen amount, grain yield, total nitrogen amount, grain number, and GPC. Moreover, previous study reported that there was a photoperiod-sensitive gene *Ppd1* on 2DS (Worland et al., 1998). A 2D locus near *Ppd1* accounting for 25% of the variation in protein content in materials grown in Mexico. The allele linked to *Ppd1* was associated with a 1.1% increase in protein concentration (Nelson et al., 2006). Notably, chromosome 2D harbors multiple quality-related traits in wheat. The *QGpc.cau-2D* locus identified in this study is spatially distinct from previously reported GPC-related QTLs on chromosome 2D. Moreover, *QGpc.cau-2D* demonstrated higher phenotypic stability and consistent detection across multiple environments compared to these known loci. These findings collectively support the conclusion that *QGpc.cau-2D* represents a novel genetic locus governing GPC in wheat. Furthermore, we developed a STARP marker within this candidate region that co-segregates with the GPC trait. This marker is expected to facilitate the selection of high-quality wheat cultivars in future breeding programs.

Within the *QGpc.cau-2D* interval, we identified nine putative candidate genes. Among these, *TraesCS2D03G0079100* encodes an ELMO domain-containing protein. Previous research has demonstrated that ELMO1 is critical for cell adhesion, suggesting functional conservation across family members (Kohorn et al., 2021). *TraesCS2D03G0080800* encode GDSL esterase/lipase. The GDSL-type esterase/lipase protein is a newly discovered lipid hydrolysis enzyme (lipolytic enzyme). These enzymes primarily modulate plant development, morphogenesis, secondary metabolite synthesis, and defense responses (Zhang et al., 2021). OsGELP34, OsGELP110 and OsGELP115 from rice were proven to regulate the pollen development (Zhang et al., 2020). *TraesCS2D03G0082600* encode NBS-LRR disease resistance protein, which have been described as key components of plant immunity responsible for pathogen recognition and triggering defense responses (Kapos et al., 2019). *TraesCS2D03G0092200* encodes nicotianamine synthase (NAS), which catalyzes the synthesis of nicotianamine (NA). This low-molecular-weight compound functions as a metal chelator in plants, with its tissue accumulation dynamically responding to metal deficiency or excess (Seregin and Kozhevnikova, 2023). *TraesCS2D03G0092700* encode 60 kDa chaperonin, which can facilitate the folding of proteins (Thirumalai and Lorimer, 2001). No evidence has demonstrated direct or indirect associations between these genes (or their homologue) and phytohormone signaling, nitrogen transport, GPC, or other quality traits. Consequently, they were excluded from further analysis.

*TraesCS2D03G0079200* encode E3 ubiquitin-protein ligase WAV3, which belongs to the RING-like zinc finger family. Previous studies have demonstrated that the U-box E3 ubiquitin ligase PUB35 negatively regulates ABA signaling through AFP1-mediated degradation of ABI5 (Du et al., 2024). Meanwhile, the radiation sensitive 23B protein modulates *Arabidopsis* root development through the E3 ubiquitin ligase EDA40 (Javed et al., 2025). *TraesCS2D03G0088900* encode ABC transporter G family member, which mediates the transport of a broad spectrum of structurally diverse compounds and is involved in important processes that influence plant fitness (Gräfe and Schmitt, 2021). Moreover, In *A. thaliana* AtABCG act as auxin and cytokinin transporters (Dhara and Raichaudhuri, 2021). *TraesCS2D03G0080700* and *TraesCS2D03G0081400* encode indole-2-one monooxygenases. These proteins feature conserved cytochrome P450 domains and are classified within the cytochrome P450 superfamily. In plants, cytochrome P450 enzymes mediate essential physiological processes, including redox reactions, metabolic regulation, and environmental stress responses (Donaldson and Luster, 1991). *CYP707A* catalyzes a crucial step in the ABA catabolic pathway, facilitating its degradation (Endo et al., 2011). *CYP735A* plays a vital role in cytokinin biosynthesis (Zürcher and Müller, 2016). Additionally, *CYP83B1* acts as a regulatory factor in auxin biosynthesis (Bak et al., 2001). Abscisic acid, auxin, and cytokinin have been closely linked to nitrogen signaling (Kiba et al., 2011). Meanwhile, plant root development influences the capacity for nitrogen acquisition from the soil (Kiba and Krapp, 2016). Plants typically maximize their growth and developmental potential to optimize reproductive output. Upon receiving grain formation signals, they redirect carbohydrates and free amino acids to developing grains for protein accumulation. GPC shows significant positive correlations with nitrogen fertilizer application, nitrogen uptake capacity, and nitrogen use efficiency (NUE) (Charmet et al., 2005; Cormier et al., 2016). In previous studies, NAC transcription factor (*NAM-B1*) that accelerates senescence and increases nutrient remobilization from leaves to developing grains, playing an essential role in regulating the content of grain protein and other micronutrients in wheat (Uauy et al., 2006). Additionally, *TaNAM-6A* has been demonstrated to be essential for nitrogen remobilization and GPC regulation in wheat (Meng et al., 2024). Collectively, we propose that the four identified genes may indirectly regulate nitrogen uptake and utilization by modulating phytohormone biosynthesis or degradation, thereby influencing GPC. Based on resequencing data and functional annotations, the nine genes mentioned above, especially *TraesCS2D03G0079200*, *TraesCS2D03G0080700*, *TraesCS2D03G0081400*, and *TraesCS2D03G0088900*, may be the candidate genes underlying the function of the *QGpc.cau-2D* locus. Current functional predictions for these genes rely principally on homology, and their precise mechanisms governing GPC necessitate experimental validation.

## 5 Conclusions

This study employed 198 RILs derived from TAA10 and XX329 to QTL mapping and candidate gene mining for GPC. It was found that three environment-stable QTLs *QGpc.cau-2D, Qgpc.cau-4D*, and *Qgpc.cau-7D* were located on chromosomes 2D, 4D, and 7D respectively, among which *QGpc.cau-2D* on chromosome 2D displayed the most significant effect on GPC. By selecting residual heterozygous lines and heterozygous recombinant plants, *QGpc.cau-2D* was fine-mapped to a physical distance of approximately 4.2 Mb between markers *Xcau-2D541* and *Xcau-2D781* on the 2DS, which was an unreported locus. Using resequencing data from XX329 and TAA10, coupled with differential expression analysis and functional annotations, four putative candidate genes within the *QGpc.cau-2D* candidate region were identified: *TraesCS2D03G0079200*, *TraesCS2D03G0080700*, *TraesCS2D03G0081400*, and *TraesCS2D03G0088900*. These loci may providing new insights into the molecular mechanisms regulating the GPC pathway.

## Data Availability

The datasets presented in this study can be found in online repositories. The names of the repository/repositories and accession number(s) can be found in the article/**Supplementary Material**.
