# Supplementary material for: Identification and characterization of QTL for grain protein content derived from the D genome of allohexaploid wheat
Source: Front Plant Sci. 2025 Dec 1;16:1711891. doi: 10.3389/fpls.2025.1711891 (PMC12702865; doi:10.3389/fpls.2025.1711891)
Supplement: Supplementary file 2 [file Table2.docx]

Supplementary Material

# Supplementary Data


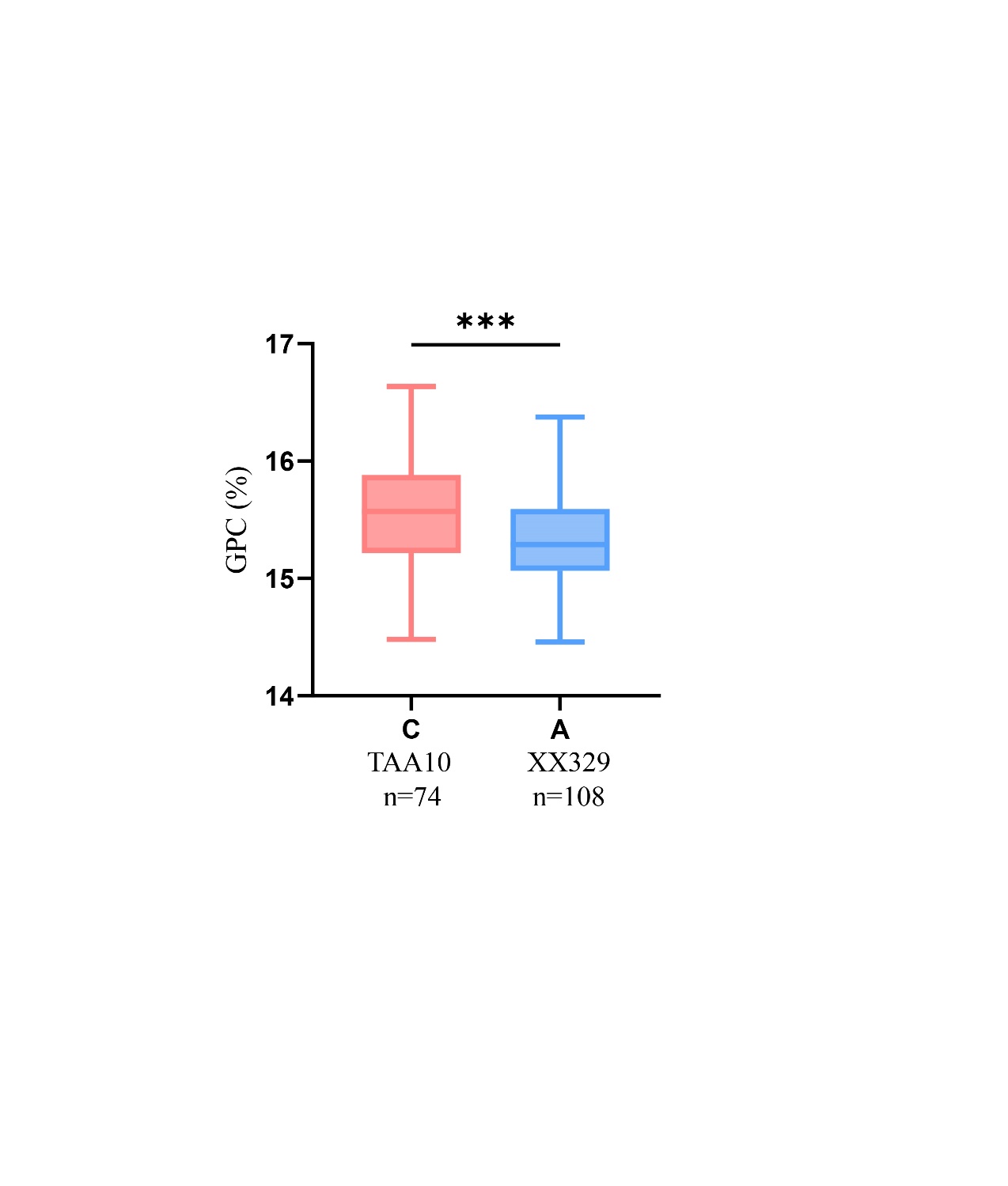


**Fig. S1** Box plots showing the effects of *QGpc.cau-2D* on GPC in the population of RILs. Asterisks indicate significant differences (*P<0.05, **P<0.01).

# Supplementary Datasets

**Table S1** Information of evaluated environments.

**Table S2** Phenotypic data of the 198 RILs under the six individual environments and the BLUP value.

**Table S3** Genotypic data of the TAA10/XX329 RIL population that used in QTL analysis.

**Table S4** An Genetic linkage map of 2D and 4D genome constructed with the TAA10/XX329 RIL population.

**Table S5** Genotypic data of the TAA10/XX329 RIL population that used in fine mapping.

**TableS6** Primers used in this study.

**Table S7** Annotated genes in the interval between the molecular markers *Xcau-2D541* and *Xcau-2D781*

**Table S8** Sequence variation analysis of putative candidate genes that are expressed in the wheat grain.
